# The Effect of Spiritual Leadership on Employee Effectiveness: An Intrinsic Motivation Perspective

**DOI:** 10.3389/fpsyg.2018.02627

**Published:** 2019-01-04

**Authors:** Minghui Wang, Tengfei Guo, Yakun Ni, Sudong Shang, Zheng Tang

**Affiliations:** ^1^School of Educational Science, Henan University, Kaifeng, China; ^2^School of Management & Marketing, University of Waikato, Hamilton, New Zealand

**Keywords:** spiritual leadership, task performance, knowledge sharing behavior, innovation behavior, ethical leadership

## Abstract

Drawing on spiritual leadership theory and intrinsic motivation theory, we proposed a homologous multilevel model to explore the effectiveness of spiritual leadership on employees’ task performance, knowledge sharing behaviors and innovation behaviors at the individual level. With questionnaires rated by 306 pairs of employees and their supervisors in 26 teams from the energy industry in mainland China, we conduct multilevel analysis to examine our hypotheses. The results show that spiritual leadership was positively related to employee task performance, knowledge sharing behaviors and innovation behavior, when we controlled for possible confounding effects of moral leadership and benevolent leadership, and ruled out alternative explanation of ethical leadership. The theoretical and practical implications are discussed.

## Introduction

Along with growing uncertainty and new challenges facing organizations in dynamic environments (Tyssen et al., 2014), an enormous number of enterprise managers focus on creating a clear organizational vision, forming favorable organizational cultures, and inspiring employees’ inner motivation to increase the competitive advantage of the organization (Chen and Li, 2013; Chen et al., 2013). One approach that embodies such management patterns is spiritual leadership, which incorporates vision, hope/faith, and altruistic love to motivate oneself and others in order to have a sense of spiritual survival (Chen et al., 2013). This leadership style points the way that could intrinsically inspire employees to work beyond role obligation for the common good of the group. Despite much attention having been drawn to the significant impact of spiritual leadership on facilitating organizational development and transformation, our knowledge is very limited regarding the effects of spiritual leadership at the individual level. In this paper, we investigate how spiritual leadership affects employee effectiveness, and simultaneously control other related leadership styles, such as moral leadership, benevolent leadership and ethical leadership.

Spiritual leadership theory was developed within the intrinsic motivation model (Fry, 2003). Intrinsic motivation refers to an inherent tendency to seek out novelty and challenges, to extend and experience one’s capacities and to learn (Ryan and Deci, 2000), which represents the prototypic manifestation of the human tendency toward learning and creativity (Ryan, 1995). Various studies have confirmed that intrinsic motivation is associated with better learning, creativity, and performance. Some studies have shown that individuals who are intrinsically motivated have more interest and confidence than those who are externally controlled, which in turn is manifested as enhanced performance and creativity (Valas and Slovik, 1993; Sheldon et al., 1997). Other studies have demonstrated that intrinsically motivated individuals engage in self-determined behaviors, such as knowledge sharing behaviors and innovative work behaviors (Devloo et al., 2015; Tangaraja et al., 2015). Finally, intrinsically motivated individuals engage in tasks primarily because the task itself is satisfying (Yousaf et al., 2015).

Furthermore, spiritual leadership theory is designed to create an intrinsically motivated, learning organization (Fry et al., 2005). Consistent with intrinsic motivation theory, spiritual leadership is considered as an effective approach to foster higher levels of organizational productivity, team creativity, and organizational learning capacity (Jurkiewicz and Giacalone, 2004; Aydin and Ceylan, 2009; Chen and Yang, 2012). Moreover, spiritual leaders are concerned with active engagement in the workplace such that people experience meaning in life, which in turn promotes followers’ growth and development. In the process of both transforming a learning organization and employee growth, intrinsically motivated followers inevitably tend to be highly efficient at completing their mission and actively engage in sharing knowledge and implement novel ideas (Andrews and Delahaye, 2000; Fraj et al., 2015). However, the relationship between spiritual leadership and task performance, innovation behaviors, and knowledge sharing behaviors are seldom explored at the individual level in existing research.

Therefore, this paper aims to investigate the effectiveness of spiritual leadership at an individual level, based on intrinsic motivation theory and spiritual leadership theory. A holistic view of leadership effectiveness looks at both the leader’s effect on followers and achievement of the goal (Reave, 2005). The current research empirically examines whether spiritual leadership positively affects two categories of employee effectiveness, follower behaviors (e.g., knowledge sharing behaviors and innovation behaviors) and execution of an assigned goal (e.g., task performance).

However, Anderson and Sun (2017) suggested that previous empirical studies on spiritual leadership did not control for other leadership styles, and it is unknown whether spiritual leadership adds predictive variance above other styles. We argue that ethical leadership can be a plausible alternative explanation when researchers explore the effectiveness of spiritual leadership. Reave (2005) suggested that spiritual leadership closely aligns with ethical leadership and requires moral character and an ethical climate, and such spiritual motives might influence someone to become an ethical leader (Brown and Treviño, 2006). Moreover, there is a great deal of overlap between the two theoretical models. Spiritual leadership theory comprises ethical aspects (e.g., ethical consideration, integrity, and trust) and demonstrates that spirituality cannot exist without ethical value (Kuhnert and Lewis, 1987). Hence, the second purpose of this paper is to rule out alternate ethical leadership explanations, focusing on whether spiritual leadership adds predictive variance above and beyond these other styles.

**FIGURE 1:**
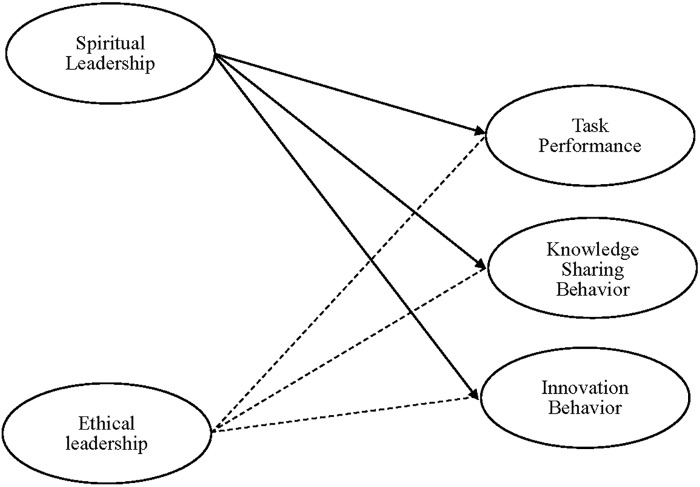
Theoretical model.

Our research provides several contributions to the extant literature. First, although previous research demonstrated that spiritual leadership was positively associated with beneficial organizational outcomes, little is known about the relationship between spiritual leadership and task performance, knowledge sharing behaviors and innovation behaviors at the individual level. Our research will provide initial evidence of how spiritual leadership is linked to positive employee behaviors and outcomes. Moreover, our study explores an alternate model (ethical leadership) as an explanation for our results while providing evidence for the robustness of the effect. By doing so, the current research enriches our understanding of spiritual leadership theory and intrinsic motivation theory, which is expected to advance relevant research and practice in the domain of spiritual leadership. The overall theoretical model is presented in Figure 1.

## Theory and Hypotheses

### Spiritual Leadership Theory

Fry (2003) incorporated spirituality, a long-neglected aspect, into leadership theories, and ultimately proposed the concept of spiritual leadership, which emphasizes intrinsically motivating one’s self and others through the leader’s values, attitudes, and behaviors. Conceptually, spiritual leadership comprises three principal components, vision, hope/faith, and altruistic love, as the leader’s values, attitudes, and behaviors, respectively. Vision refers to a meaningful future, causing employees to feel intrinsic self-value and life purpose. Hope/faith reflects the leader’s confidence in the achievability of the vision, high levels of which can inspire subordinates to accomplish the organizational mission. Altruistic love exhibits a series of leader behaviors valuing mutual care and respect and producing a sense of being understood and appreciated by organizational members, based on which a favorable organizational culture is likely to be forged. Vision in the spiritual leadership model gives intrinsic purpose to life (Chen and Yang, 2012) and is spiritually grounded when employees have a sense of hope/faith that the common vision will engage them in achieving future goals (Fry et al., 2005; Fry and Cohen, 2009). At its best, this feeling is the intrinsic reward for employees to create firm beliefs and encourage the pursuit of a meaningful organizational vision (Chen and Yang, 2012).

Although spiritual leadership theory is deeply rooted in Western culture, several scholars have argued for the transportability of the leadership construct and organizational practice to the Confucian cultural context. For instance, high levels of spirituality in leaders is positively associated with the achievement of organizational goals in South Korea (Kang et al., 2017). Furthermore, spiritual leadership provides a unique approach to protect company resources and decrease subordinates’ wrongdoing in the Confucianism context (Wang et al., 2017).

### Spiritual Leadership and Task Performance

Task performance involves a specific pattern of behaviors that orient toward completing a work task and make a unique contribution to supervisor’s judgment of an employee’s overall worth to the organization (Motowidlo and Van Scotter, 1994; Conway, 1999; Johnson, 2001). There are reasons for the positive relationships between spiritual leadership and individual task performance. First, spiritual leadership is viewed as an effective way to fuel employees’ intrinsic motivation. Spiritual leadership not only meets the psychological needs of both leaders and followers (Guillén et al., 2015) but also taps into fundamental needs for spiritual survival, which include spirituality values and management practices, such as inspiring people to seek interesting and meaningful work (Fry, 2003). Both interest and basic psychological needs are critical, defining characteristics of intrinsic motivation, and intrinsically motivated behaviors are a function of psychological needs and interest, which are manifested through autonomy, competence, and relatedness in the workplace (Deci and Ryan, 2000; Ryan and Deci, 2000). Additionally, intrinsic motivation will more likely flourish in contexts characterized by a sense warmth and caring (Deci and Ryan, 2000). The purpose of spiritual leadership is to intrinsically motivate followers through practicing spirituality values and exhibiting altruistic love in the workplace. Ultimately, the goal is to foster high-level productivity (Fry, 2003; Fry et al., 2005; Fry and Cohen, 2009). Various studies have suggested that intrinsic motivation is associated with better performance and learning (Grant, 2008; Cerasoli and Ford, 2014; Menges et al., 2017).

Second, followers of leaders with spiritual leadership are more likely to have better performance because of the common and clear vision. Spiritual leadership entails motivating followers by articulating a long-term challenge and different future. Clear and sufficiently challenging goals are more likely to improve an individual’s task performance (Locke et al., 1981). Specific and challenging goals lead to higher output than those assigned no specific goals (Locke et al., 1981; Corgnet et al., 2015). Spiritual leadership delivers faith/hope both in spirituality-grounded vision and the process of creating vision for followers. As a role model, the confident attitude inspires followers to display their tenacity and pursue excellent performance by doing their best in achieving challenging tasks (Yang et al., 2017). To summarize, we hypothesize the following relationship:

Hypothesis 1: Spiritual leadership will be positively associated with employees’ task performance.

### Spiritual Leadership and Knowledge Sharing Behaviors

Knowledge sharing behaviors are defined as a series of actions in which individuals disseminate and diffuse valuable information with others within organizations (Bartol and Srivastava, 2002). These behaviors represent a process of mutually exchanging relevant information, and it implies synergistic collaboration among individuals who work toward a common goal (Boland and Tenkasi, 1995). Empirical studies have identified that leadership is an important factor that influences knowledge sharing attitudes and behaviors (Gagné, 2009). Consistently, Bock and Kim (2002) found that having leaders who expected to improve subordinates’ relationships and to recognize employees’ contribution to organizational performance was positively related to sharing behaviors. Spiritual leadership is a type of leadership characterized by defending integrity, goodness, teamwork, knowing, wholeness, and interconnectedness (Aydin and Ceylan, 2009). The purpose of spiritual leadership is to paint a desirable vision and value congruence across the strategic plan (Fry, 2003), and this shared vision vividly portrays a picture that followers should solve problems and share valuable knowledge to fulfill a common goal, when encountering complex challenges. Sharing professional knowledge is viewed as a personally worthwhile realization for employees who have internalized the organizational vision into their value systems (De Vries et al., 2006).

Additionally, knowledge sharing behaviors are a kind of intentional behaviors that share similarity with various voluntary behaviors (Frey, 1993), such as prosocial behavior and organizational citizenship behavior. This kind of behavior is likely to be motivated by intrinsic motivation (Wang and Hou, 2015). Various research has suggested that intrinsic motivation was associated with effective information sharing and corporate ability (Brammer et al., 2015; Razmerita et al., 2016). Deriving from intrinsic enjoyment of helping others, altruism is significantly intrinsically motivated (Hung et al., 2011). Similarly, recent research has confirmed that an individual’s altruistic intention predicts knowledge sharing behaviors (Matzler et al., 2008) because it is enjoyable or it is personally meaningful for them. Spiritual leadership theory is fundamentally rooted in the intrinsic motivation model, consistent with the intrinsic motivation of enjoying helping others, which establishes an organizational culture encompassing the value of altruistic love. Thus, we submit that spiritual leadership likely promotes followers to exhibit knowledge sharing behaviors.

Although to date this proposition remains untested, a study by Aydin and Ceylan (2009) provided some support for the influence of spiritual leadership on knowledge sharing behavior. In the study, they found that organizational learning capacity was significantly positively correlated with each of the spiritual leadership dimensions. Moreover, the extent of individuals acquiring the knowledge and sharing the information are key dimensions of organizational learning capacity. We therefore hypothesized the following:

Hypothesis 2: Spiritual leadership will be positively related to followers’ knowledge sharing behaviors.

### Spiritual Leadership and Innovation Behaviors

Innovation behaviors refer to the intentional creation, introduction and application of new ideas within a work role, group or organization (De Jong and Den Hartog, 2010). Researchers have regarded leadership as pivotal in innovative processes (Rosing et al., 2011; Donate and de Pablo, 2015), but to the best of our knowledge, there is still a scarcity of research that demonstrates a clear link between spiritual leadership and innovation behaviors. Organizations that are rich in spirituality generate creativity among team members, since spirituality is a key element of creativity and innovation processes. Spiritual leadership is an effective approach to nourishing spirituality within the workplace (Fry, 2008), ultimately facilitating followers’ innovation behaviors. Specifically, spiritual leaders value followers’ meaningfulness at work and motivate them to go beyond the call of duty, such that followers will have a sense of self-transcendence. This experience is an important antecedent of innovation behaviors (Jung et al., 2003). In addition, spiritual leaders are characterized as having integrity, honesty, altruism and genuine care for others (Fry, 2003; Reave, 2005). Those characteristics are typically embedded in spiritual leaders who are preoccupied with followers’ development. The focus on followers’ development is a result of meeting their foundational psychological need for safety. Followers who see themselves as significant in the workplace and have a strong sense of psychological safety will be more likely to generate new ideas and experiment with the ideas (Hogan and Coote, 2014).

Drawing from spiritual leadership theory, spiritual leadership develops a compelling vision that articulates the road to fulfilling followers’ ideas in the workplace since providing a shared vision is believed to inspire innovation behaviors (Weng et al., 2015). In the vision-creating process, spiritual leadership entails subordination of their own goals and followers’ most desirable elements for the common greater future of the organization while also keeping followers’ trust and belief in the organization’s vision through fueling their hope/faith. Spiritual leaders directly effect cognitive-based trust and affect-based trust through the process of identifying vision and motivating faith/hope, which in turn increases followers’ intrinsic motivation to implement creative ideas to pursue organizational vision (Yoshida et al., 2014). In addition, spiritual leaders engage in establishing a sustained organizational culture based on altruistic love (Fry, 2003). Such a culture focuses on followers’ growth and inspires positive emotions (Yoshida et al., 2014). Specifically, positive emotion can encourage followers to explore new ideas in problem-solving (Joiner et al., 2001). Numerous studies have suggested that positive emotions inspire innovative behaviors. Individuals who experience positive emotions such as joy and being respected tend to pursue novel, problem-solving methods (Rose et al., 2016). Therefore, we proposed the following:

Hypothesis 3: Spiritual leadership is positively related to followers’ innovation behaviors.

## Materials and Methods

### Participants and Procedures

In cooperation with several energy companies located in Northern China, participants in the current study were recruited through election of “Outstanding Annual Supervisors” within the energy industry, who were formal employees of two types of enterprises including state-owned enterprises and non-state-owned enterprises. The main selection standards for “Annual Outstanding Supervisors” included (1) those with high ethical standards, (2) those equipped with leadership competency based on post requirements, and (3) outstanding performance of departments where the candidate belonged. A necessary section of the election program was the candidate’s subordinates assessing the candidate’s leadership behaviors. Thus, it was a unique opportunity to obtain multisource data from each of the participating supervisors. The questionnaire was designed to separate into two sets: one set of supervisors (candidates) and the other set for immediate subordinates.

After getting approval from the senior management department, we collected data at two different time points with a 4-month interval. At the first timepoint (T1), the candidates’ immediate subordinates rated their perception of supervisors’ sipritual leadership, and control variables (e.g., moral leadership,benovent leadership, type of enterprise and demographics) were measured at this time. At the second timepoint (T2), we distributed ethical leadership questionnaires to the same participants at T1, and supervisors were asked to rate their subordinates’ task performance, knowledge sharing behaviors and innovative behaviors. Before the participants registered for the survey, they were assured of anonymity and confidentiality. Overall, by matching company’s identification number for subordinates with supervisors, and eliminating the illegible and unmatched questionnaires, the final sample included a total of 306 pairs on 26 teams. Team features are: the range of team members from 5 to 21 (the average team member = 11.85); 17 out of 26 teams belong to state-owned enterprises (65.39%); and 9 teams belong to non-state-owned enterprises (34.61%).

In addition, among supervisors (*n* = 26, with an effective response rate of 92.85%), 92.3% were male (*n* = 24), and they were mostly aged 35–45 years (*n* = 21, 80.70%). Moreover, approximately 76.92% of respondents reported that they had more than 8 years’ work experience (*n* = 20), and over 69% of respondents had completed a bachelor’s degree (*n* = 18). Of the 280 subordinates (with an effective response rate of 89.74%), 87.5% were male (*n* = 245), and most were aged 26–35 years (*n* = 253, 90.35%). They had an average organizational tenure of 4 years, and 70.71% had a junior college degree (*n* = 198).

### Measures

#### Spiritual Leadership

Spiritual leadership was assessed by the Spiritual Leadership Questionnaire of Fry (2008) at T1. It was a multidimensional construct including three portions: (a) 5 items for Vision (α = 0.92), (b) 5 items for Hope/Faith (α = 0.92), (c) 7 items for Altruistic Love (α = 0.85). Example items include the following: “I understand and am committed to my organization’s vision.” (Vision); “set challenging goals for my work because I have faith in my organization and want us to succeed.” (Hope/Faith); “The leaders in my organization dare to stand up for their people.” (Altruistic Love). The reliability of the scale was 0.93.

#### Ethical Leadership

Supervisors’ ethical leadership was measured by Ethical Leadership Questionnaire with a 10-item scale developed by Brown et al. (2005) at T2. Example items are “my supervisor talks about the importance of ethics” and “my supervisor sets an example of how to do things the right way regarding ethics.” Response options ranged from 1, “strongly disagree” to 7, “strongly agree.” Cronbach’s alpha of the scale was 0.74.

#### Moral Leadership and Benevolent Leadership

Paternalistic leadership was assessed by the Paternalistic Leadership Questionnaire of Cheng et al. (2004) at T1. It was a multidimensional construct including three portions: (a) Moral Leadership (α = 0.84), (b) Benevolent leadership (α = 0.78), (c)Authoritarian Leadership (α = 0.87). Example items are “my supervisor is like a family member when he/she gets along with us” and “my supervisor does not take advantage of me for personal gain” Response options ranged from 1, “strongly disagree” to 5, “strongly agree.” Cronbach’s alpha of the scale was 0.83.

#### Task Performance

We measured task performance by using a 4-item scale developed by Fan and Zheng (1997) at T2. This scale is the most common measurement of task performance (Cronbach’s alpha = 0.88) in Chinese context. Items were rated on a 5-point scale ranging from 1 (totally disagree) to 5 (totally agree). Sample items include “One of the best employees in the department (organization)”.

#### Knowledge Sharing Behavior

We measured knowledge sharing behavior by using a 5-item scale developed by Ruan (1997) in Chinese context at T2. Cronbach’s alpha of this overall scale is 0.89. Items were rated on a 5-point scale ranging from 1 (totally disagree) to 5 (totally agree). Sample items include “Often take the initiative to share his knowledge with his colleagues.”

#### Innovation Behavior

We measured knowledge innovation behavior by using a 6-item scale developed by Scott and Bruce (1994) at T2. Cronbach’s alpha of this overall scale is 0.88. Items were rated on a 5-point scale ranging from 1 (totally disagree) to 5 (totally agree). Sample items include “Often take the initiative to share his knowledge with his colleagues.”

#### Control Variables

Previous studies have found that paternalistic leadership has deep cultural roots in China. Consistent with the core values of Confucianism, paternalistic leadership emphasizes constraining one’s behavior with moral norms (Cheng et al., 2014). An enormous amount of research on the outcomes of paternalistic leadership indicates that employee performance was significantly affected by moral leadership and benevolent leadership rather than by authoritarian leadership (Zhang et al., 2015). Furthermore, benevolent leadership and moral leadership were strongly related to positive outcomes, such as individual sharing behaviors and organizational citizenship behaviors. Accordingly, several control variables (e.g., moral leadership and benevolent leadership) were introduced in our analysis and expect to minimize the effects of other exogenous variables. Besides, participants in our research are from state-owned enterprises and non-state-owned enterprises. Research has shown that the extent of the application of leadership effectiveness may vary in the different types of enterprises (Liu et al., 2003; Hooijberg and Choi, 2007). Thus, this team feature was also introduced in our analysis.

#### Analyses

First, we employed Pearson’s correlational tests and independent samples t-tests to examine the correlations among study variables and whether they differed by demographic variables (i.e., age, work tenure, gender, educational level and type of enterprise). The results of correlational tests are presented in Table 1. Additionally, independent samples *t*-tests indicated that spiritual leadership (*t* = -2.67, *p* < 0.01) and ethical leadership (*t* = 14.29, *p* < 0.01) were significantly different by type of enterprise. Spiritual leadership characterized participants (*M* = 4.33, *SD* = 0.58) in state-owned enterprises more than it did for participants from non-state owned enterprises (*M* = 4.13, *SD* = 0.089). Moreover, the score on ethical leadership was higher among state-owned enterprises (*M* = 3.79, *SD* = 0.678) than that among non-state-owned enterprises (*M* = 3.97, *SD* = 0.90). Subsequently, we used exploratory factor analysis with parallel analysis and multiple-level confirmatory factor analysis to test discrimination validity among spiritual leadership, ethical leadership and control variables (i.e., benevolent leadership, moral leadership). Exploratory factor analysis is an approach based on sample data features to reduce dimensions to obtain common factors. Considering the concept of spiritual leadership to be a multidimensional structure, the common differential factors are more likely mixed sub dimensions of concepts instead of the sole concept. We require sufficient theoretical evidence to justify the conceptual distinctiveness of focal variables. Moreover, owing to the fact that sample data were collected from various work teams and considering the nested nature of the data, multilevel confirmatory factor analysis was conducted. Table 2 presents multilevel confirmatory factor analysis results. Afterwards, to examine the effectiveness of spiritual leadership on performance outcomes while ruling out alternative ethical leadership explanations, we used multilevel modeling analysis to test our hypotheses.

**Table 1:** Descriptive statistics and correlations among all variables.

Variable	1	2	3	4	5	6	7	8	9	10	11	12
(1) Gender	1											
(2) Age	-0.10	1										
(3) Educational level	0.09	-0.33**	1									
(4) Work tenure	-0.18**	0.50**	-0.32**	1								
(5) Enterprise type	0.24**	-0.11*	-0.01	-0.43**	1							
(6) Spiritual leadership	0.04	-0.02	0.00	-0.07	0.15**	(0.93)						
(7) Ethical leadership	-0.19**	0.04	0.02	0.25**	-0.63**	0.31**	(0.74)					
(8) Benevolent leadership	-0.06	-0.02	0.08	0.06	-0.05	0.06	0.13*	(0.78)				
(9) Moral leadership	0.06	-0.03	0.17**	-0.07	0.06	0.11*	-0.03	0.19**	(0.84)			
(10) Task performance	0.07	-0.05	0.16**	-0.08	-0.07	0.45**	0.28**	0.10	0.01	(0.88)		
(11) Knowledge sharing behavior	0.01	-0.02	0.11	-0.01	-0.05	0.61**	0.41**	0.12*	0.11	0.48**	(0.89)	
(12) Innovation behavior	0.01	0.04	0.02	-0.03	0.00	0.63**	0.34**	0.06	0.08	0.49**	0.69**	(0.88)
Mean	1.46	3.28	2.40	3.34	1.33	4.23	3.85	2.89	3.06	4.17	4.09	4.08
*SD*	0.51	0.66	0.87	1.17	0.47	0.49	0.80	0.55	0.74	0.64	0.64	0.59


*Cronbach’s alpha coefficients are in parentheses on the diagonal. ^∗^p < 0.05 and ^∗∗^p < 0.01.*

**Table 2:** Summary of model fit for the four-factor model (*N* = 306).

Structure	χ^2^	df	χ^2^/df	CFI	TLI	RMSEA	SRMR
Conventional CFA	233.32	129	1.80	0.92	0.90	0.07	0.07
Pooled-within model	643.66	130	4.95	0.66	0.60	0.11	0.10
MCFA	336.88	292	1.15	0.96	0.95	0.03	IL:0.07 TL:0.30


*IL, individual level; TL, team level.*

## Results

### Description

Table 1 shows the mean, standard deviation, and correlations for each of the constructs and demographic and control variables. It was found that spiritual leadership was significantly and positively correlated with employees’ task performance (*r* = 0.45, *p* < 0.01), knowledge sharing behaviors (*r* = 0.61, *p* < 0.01) and innovation behaviors (*r* = 0.63, *p* < 0.01); these results provided initial support for H1, H2, and H3, respectively. Ethical leadership was significantly and positively correlated with task performance (*r* = 0.28, *p* < 0.01), knowledge sharing behaviors (*r* = 0.41, *p* < 0.01) and innovation behaviors (*r* = 0.34, *p* < 0.01). Control variables including benevolent leadership and moral leadership were also significantly and positively correlated with task performance, knowledge sharing behaviors and innovation behaviors.

### Conceptual Discrimination Validity

We conducted an exploratory factor analysis with parallel analysis and multilevel factor analysis with maximum likelihood estimation using MPLUS 7.0 to examine whether employees’ scores on measures (i.e., spiritual leadership, ethical leadership, benevolent leadership, and moral leadership) captured distinctive constructs. Parallel analysis is one of the most accurate factor retention methods in exploratory factor analysis (Hayton et al., 2004). Specifically, according to Reise et al. (2000), exploratory factor analysis with parallel analysis consists three main steps: first, generating a set of random data matrix that contains the same number of indices and sample size as observation data, second, the eigenvalues of this set of random data are calculated, and the average value of these eigenvalues is obtained, and third, the curve of eigenvalues and scree plot of observation data are compared to determine the focus of the two curves, which is the specific number of common factors that should be retained. In the current study, parallel analysis suggested that six factors with eigenvalues over 2.21 should be retained. According to the conceptual model, except for spiritual leadership as a multidimensional structure that has three sub dimensions, the other three factors have single dimension structures. Thus far, the discrimination validity of relevant core variables in this study was partially supported. Nonetheless, compared with pure date-based analysis, it is necessary to conduct a theory-based test to examine the conceptual discrimination validity.

Given that our hypotheses were proposed at an individual level, in multilevel confirmatory factor analysis, we treated all four focal variables (i.e., spiritual leadership, ethical leadership, benevolent leadership, and moral leadership) as individual-level factors while their team level variance was controlled. To control for measurement error and improve the sample size to parameter ratio, observed indicators were formed into several parcels by item parceling, and constructs were modeled using latent variables. We formed four different constrained measurement models with all observation indicators comparing a four-factor model to examine the distinctiveness of the focal concept. The results (see Table 2) of multilevel confirmatory factor analysis showed that the fit indices of the four-factor model fit the data well. Furthermore, the four-factor model fit the data better than all constrained measurement models [336.88 < Δχ(Δdf = 6) < 539.12, *p* < 0.01]. As noted above, the distinction among the focal variable constructs was verified by the mutual complementary empirical approaches of data-based analysis (exploratory factor analysis) and theory-based analysis (confirmatory factor analysis). Thus, the result provided support for construct distinction.

### Hypotheses Test

With the nested nature of the data, we employed a series of multilevel structural equation models (Level 1: individual level; Level 2: team level) to test the effectiveness of spiritual leadership on employee performance outcomes (e.g., task performance, knowledge sharing behavior and innovation behavior), and observed indicators were formed into several parcels by item parceling. First, we estimated a multilevel model that specified the level 1 fixed spiritual leadership on task performance (model 1a) and then added ethical leadership into the model (model 1b). Benevolent leadership, moral leadership and the type of enterprises were also controlled in model 1a and model 1b. At level 2, all level 2 intercepts of level 1 focal variables (i.e., benevolent leadership, moral leadership, spiritual leadership and ethical leadership) were set to correlate with each other freely. All exogenous variables were grand-mean centered. The results showed that controlling for benevolent leadership and moral leadership, spiritual leadership was significantly related to task performance (β = 0.34, *p* < 0.01), and after entering ethical leadership, spiritual leadership was significantly related to task performance (β = 0.33, *p* < 0.01), but ethical leadership was insignificantly related to task performance (β = 0.04, *p* > 0.05). Subsequently, we tested the effectiveness of spiritual leadership on knowledge sharing behaviors. At level 1, spiritual leadership was fixed on knowledge sharing behavior along with control variables (model 2a), and then ethical leadership was entered to predict knowledge sharing behavior (model 2b). At level 2, all focal variables from level 1 were freely correlated with each other. The results of model 2a showed that spiritual leadership was significantly related to knowledge sharing behavior (β = 0.27, *p* < 0.01), and in model 2b, the effect of spiritual leadership on knowledge sharing behavior was significant. After ruling out the alternative explanation of ethical leadership, the effect of spiritual leadership on knowledge sharing behavior decreased from 0.21 to 0.10. Ethical leadership was significantly related to knowledge sharing behavior (β = 0.32, *p* < 0.01). Lastly, adopting the same model setup, hypothesis 3 was tested, and the results showed that spiritual leadership was significantly related to innovation behaviors both in model 3a (controlling for benevolent leadership and moral leadership, β = 0.40, *p* < 0.01) and model 3b (ruling out the alternative explanation of ethical leadership, β = 0.40, *p* < 0.01). Therefore, H1-H3 were fully supported (presented in Table 3). Taken as a whole, these results increase our confidence in the general pattern of results seen in Table 3 by both ruling out an alternative explanation and exogenous confounders, providing direct evidence in support of our proposed theoretical explanation.

**Table 3:** Effectiveness of spiritual leadership and ethical leadership on employee outcomes (*N* = 306).

	Task performance				Knowledge sharing behavior				Innovation behavior			

	Mold 1a		Mold 1b		Mold 2a		Mold 2b		Mold 3a		Mold 3b	
	Estimated effect	*SE*	Estimated effect	*SE*	Estimated effect	*SE*	Estimated effect	*SE*	Estimated effect	*SE*	Estimated effect	*SE*
TE	0.04	0.04	0.04	0.05	0.06	0.05	0.04	0.05	0.08	0.07	0.07	0.06
BL	0.08	0.06	0.07	0.06	0.10^∗^	0.05	0.07	0.04	0.45^∗∗^	0.11	0.34^∗^	0.15
ML	0.02	0.06	0.01	0.06	0.14	0.09	0.11	0.09	0.14	0.07	0.13	0.12
SL	0.34^∗∗^	0.06	0.33^∗∗^	0.06	0.27^∗∗^	0.04	0.10^∗∗^	0.03	0.40^∗∗^	0.07	0.40^∗∗^	0.07
EL			0.04	0.08			0.32^∗∗^	0.06			0.02	0.10


*TE, type of enterprise; BL, benevolent leadership; ML, moral leadership; SL, spiritual leadership; BL, ethical leadership. ^∗^p < 0.05, ^∗∗^p < 0.01.*

## Discussion

As an organization’s relative advantage rests on accomplishing the assigned task, managing knowledge and implementing novel ideas, research must identify ways to intrinsically inspire employee’s task performance, knowledge sharing behaviors and innovation behaviors. This study addresses this challenge and illustrates the effective leadership activation process by which spiritual leaders achieve those outcomes. Extending the Western leadership concept to the Chinese context, we suggested that spiritual leaders positively promote important individual organizational behaviors. Our study findings show that leaders’ spirituality is an important resource to intrinsically stimulate employees’ task performance, knowledge sharing behaviors and innovation behaviors perspective in Chinese culture.

### Theoretical Implications

Spiritual leadership has emerged as a new leadership style that is vital to facilitating followers’ organizational performance (Faro et al., 2014). Our study provides early evidence of the importance of spiritual leadership in promoting follower task performance, knowledge sharing behaviors and innovation behaviors by virtue of ruling out ethical leadership as an alternative explanation, and simultaneously taking into account possible confounding effects of moral leadership and benevolent leadership. This study is probably our most significant contribution to the spiritual leadership literature. By extending our application of these processes to spiritual leadership at a personal level, we illustrate the substantial robustness of this theory for understanding spiritual leadership. To the best of our knowledge, this study is the first to investigate spiritual leadership as a more significant predictor of followers’ effectiveness compared to other leadership approaches. This study echoes the claim that empirical investigation of discrimination and incremental effects of spiritual leadership and other related leadership theories is necessary (Fry et al., 2011) and provides an answer to the question posed by Anderson and Sun (2017), which is whether spiritual leadership adds predictive variance above and beyond these other styles.

Spiritual leadership has significant effects on followers’ effectiveness when we controlled ethical leadership, benevolent leadership and moral leadership. Based on current conceptualization, spiritual leadership not only is the highest level of ethical character but also encompasses value-based and spirituality-based aspects (Chen and Yang, 2012; Hackett and Wang, 2012). Fry (2003) explained two elements necessary for spiritual leadership: creating a vision to articulate where to be, and establishing a favorable organizational culture based on altruistic love producing a sense of being accepted and appreciated. Spiritual leadership emphasizes leaders’ unconditional care and love for others, and also takes into account individual growth and development. Followers in workplaces that advocate altruistic love are more likely to feel psychologically safe to share knowledge and skills, and then to generate and implement novel ideas. On the other hand, there is a high degree of consensus among practitioners and scholars that a vision is important to guide and motivate employees (Fry et al., 2017). Vision in the spiritual leadership model gives intrinsic meaning and purpose to life and is spiritually grounded (Fry, 2003). A generally accepted and transcendent vision motivate and inspire followers to improve their performance (Fry et al., 2011) and promote creative ideas (Parameshwar, 2005). Moreover, hope/faith in organizations’ vision keeps followers looking forward to the future and provides the desire and positive expectation that fuels effort to pursue the vision (Fry et al., 2017). Thus, spiritual leadership is positively associated with task performance, knowledge sharing behaviors and innovation behaviors.

These results are consistent with intrinsic motivation theory. Defined as a principal source of enjoyment and vitality throughout life (Ryan and Deci, 2000), intrinsic motivation refers to engagement with activities that the individual finds interesting and enjoyable in and of themselsves (Deci and Ryan, 2000) rather than through separable consequences such as rewards and recognition (Presslee et al., 2013). Numerous studies have confirmed that intrinsic motivation is associated with knowledge sharing, performance, and innovative work behaviors (Presslee et al., 2013; Wang and Hou, 2015), which are not only all expected external and tangible rewards but explicit discipline diminishes intrinsic motivation (Ryan and Deci, 2000). Spiritual leadership rooted in intrinsic motivation theory, comprising values, attitude and behaviors that are necessary to intrinsically motivate followers toward purpose in the workplace (Fry, 2003), intrinsically motivates followers to do something significant, such as better task performance, sharing knowledge and implementing novel ideas.

Our research also extends previous knowledge about the positive relationship between spiritual leadership and employee effectiveness. Drawing on spiritual leadership theory and an intrinsic motivation perspective, we found that spiritual leadership was positively related to employee task performance, knowledge sharing behaviors and innovation behaviors. These results were consistent with previous findings (Jurkiewicz and Giacalone, 2004; Aydin and Ceylan, 2009; Chen et al., 2013). However, most empirical support for spiritual leadership has focused more attention to organizational outcomes, such organizational productivity and commitment, and as an effective approach for organizational transformation (Fry et al., 2005; Fry, 2008), but our study renders more support for applying the theory at the individual level.

### Practical Implications

Our study advances the idea that it is important to practice spiritual leadership in order to support employees’ task performance, knowledge sharing behaviors, and innovation behaviors. First, it is recommended that organizations should emphasize that they value spiritual leadership during the recruiting process, and select individuals with characteristics and values that predispose them to spiritual leadership. Moreover, the organization should execute a comprehensive assessment process to identify candidates who have high spiritual intensity.

Second, organizations should organize leadership training programs that are based on approaches designed to develop individual spirituality. These organizations should form an organizational culture embedded with altruistic love, and an effective and generally accepted vision developed through mutual communication. In the vision, hope/faith can be delivered in order to intrinsically inspire leaders and followers to persevere achieve challenging goals. Organizations should regularly assess whether employees understand the vision and feel genuinely cared for by leaders.

Furthermore, organizations should deepen and improve the understanding of the connotation and effects of spiritual leadership, by regularly appraising their leaders’ behaviors through 360° feedback evaluation, and by frequently discussing the requirements for spiritual leaders in competency model building, qualification management system and management cadre assessment. This process will promote leaders to transform into spiritual leaders through organizational requirements.

### Limitations and Future Research

Despite these contributions, some limitations in our work should be noted that may shed light on future research directions. First, we tested our hypotheses while controlling for possible confounding effects of moral leadership, benevolent leadership and ethical leadership. However, it is still questionable whether the effect of spiritual leadership on employee effectiveness can be generalized to other samples because we collected data solely in China and specifically in the energy industry. Data collected from multiple countries and industries would increase the generalizability of our findings. Thus, we expect future research to validate our findings with samples from other industries and countries.

Second, a limitation of our study is we did not test the potential mediating variables and conditional effects among independent variables and employee effectiveness variables. A mediator variable can serve to clarify the nature of relationship between independent variable and dependent variable (Hayes and Scharkow, 2013). In our research, there are significant direct effects of spiritual leadership but not ethical leadership on task performance and innovation behaviors. Theoretically, scholars have contended that ethical principles are a key attribute for spiritual leadership (Brown and Treviño, 2006). Therefore, according to Baron and Kenny (1986), it is valuable to investigate the mediating function of spiritual leadership on the relationship of ethical leadership with task performance and innovation behaviors in future studies.

Third, a limitation of our study is that we did not further examine the effects of sub-dimensions of spiritual leadership on employee effectiveness. Spiritual leadership corporates three interacting dimensions, vision, hope/faith, and altruistic love (Fry, 2003). Although previous studies have demonstrated that leaders’ altruistic love and a compelling spiritually grounded vision are the key dimensions of spiritual leadership that enhance spiritual well-being in organizations (Fry et al., 2011; Anderson and Sun, 2017). To the best of our knowledge, a scarcity of research has demonstrated different dimensions of spiritual leadership may have different effects on organizational and individual outcomes. Therefore, it is valuable to investigate the effects of sub-dimension of spiritual leadership on individual and organizational outcomes in future studies to enrich our understanding of this leadership style.

Despite these limitations, this study makes several important contributions. To our knowledge, the current study represents the first attempt to examine the link between spiritual leadership and employee effectiveness outcomes by ruling out the compounding effects of ethical leadership, moral leadership and benevolent leadership in one study. This study adds to a burgeoning body of research on leaders’ spirituality, indicating the importance of leaders’ spirituality in nurturing employee effectiveness.

## Ethics Statement

This study was carried out in accordance with the recommendations of ethics committee of ethical committee of Henan University with written informed consent from all subjects. All subjects gave written informed consent in accordance with the Declaration of Helsinki. The protocol was approved by the ethical committee of Henan University.

## Author Contributions

TG contributed to developing the theoretical framework, data analysis, organization, and overall writing of the paper. MW contributed to the editing and organization of the paper as well as the overall design. YN and ZT contributed to the design, data analysis, and editing of the paper. SS and MW were concerned with drafting the work and revising it critically.

## Conflict of Interest Statement

The authors declare that the research was conducted in the absence of any commercial or financial relationships that could be construed as a potential conflict of interest.
